# Regional differences and dynamic evolution of high-quality development in service industry: A case study of the Chengdu-Chongqing economic circle

**DOI:** 10.1371/journal.pone.0297755

**Published:** 2024-03-01

**Authors:** Zhixia Wu, Xiazhong Zheng, Yijun Chen, Shan Huang, Chenfei Duan, Wenli Hu

**Affiliations:** 1 Hubei Key Laboratory of Construction and Management in Hydropower Engineering, China Three Gorges University, Yichang, China; 2 College of Hydraulic & Environmental Engineering, China Three Gorges University, Yichang, China; 3 College of Management, Sichuan University of Science & Engineering, Zigong, China; 4 College of Architecture and Urban-Rural Planning, Sichuan Agricultural University, Dujiangyan, China; 5 College of Business Administration, Rajamangala University of Technology Thanyaburi, Bangkok, Thailand; Sichuan University, CHINA

## Abstract

The high-quality development of service industry has become an important engine for promoting sustainable economic development. This paper first constructed the evaluation index system of high-quality development of service industry, based on panel data from 2005 to 2020. Second, Kernel density, Markov chain and Dagum Gini coefficient were used to represent the regional differences and dynamic evolution of service industry, and the Koo method was used to explore the characteristics of spatial agglomeration. Finally, social network analysis was used to identify core indicators. The study found that: (1) From 2005 to 2020, the overall level of service industry first decreases and then increases, with Chengdu and Chongqing leading other cities. (2) The development of service industry in the CCEC has large spatial differences, mainly due to inter-regional differences. (3) The level of spatial agglomeration is less variable, with high agglomeration mainly in Chengdu. (4) Indicators such as the level of human capital are the core factors of its high-quality development. This study is of great theoretical and practical significance for the optimization and upgrading of service industry in the CCEC and the synergetic development of the region.

## Introduction

In the post-crisis era, high-quality economic development has become an important issue in promoting regional revitalization and sustainable development [1–4], and has attracted much attention from countries around the world [5]. With the development of a new round of technological revolution and the optimization of industrial structure, the transformation from ’industrial economy’ to ’service economy’ is accelerating [6]. The service industry, as one of the important industries to enhance regional economic resilience [3], has now entered a new historical period [7–9]. However, many countries in the world are now in an important period of transformation and upgrading of service industry. The service industry is characterized by differentiated development [3,10–12]. In addition, urban agglomeration, as one of the most active forms of leading the world’s economic development, is an important form of regional economic development [13,14]. However, there is obvious heterogeneity in the development of urban agglomerations. Especially for developing countries such as China, some urban agglomerations that are still in the process of development still lack specific explorations in service industry related research. Therefore, from the perspective of urban agglomerations, exploring the high-quality development of service industry can help promote its sustainable development, and then promote the sustainable development of the regional economy.

The service industry almost runs through all the intermediate links of production and life, involving many sectors [15]. Such a nature determines that service industry cannot achieve high-quality development in isolation [1]. And the urban agglomeration, as an important carrier of regional development [16], is conducive to the coordination and complementarity of the regional service industry [17]. The Chengdu-Chongqing Economic Circle (CCEC) is the country’s fourth growth pole after the Yangtze River Delta, Guangdong-Hong Kong-Macao, and Beijing-Tianjin-Hebei [18–20], which has the important functions of economic radiation and opening up to the outside world. As the largest economic circle in southwest China, it has experienced rapid economic development in recent years [21,22]. However, there is still a large gap compared to the global developed economies. Although there is a certain research base on the high-quality development of service industry, the research perspectives are mostly focused on the national or provincial level [2,23,24]. And there is a lack of analysis at the city level, as well as ignoring the coordinated relationship and regional differences of multiple cities [1]. Therefore, measuring regional differences in the CCEC can help enrich the existing research. It also helps to understand the current situation of regional service industry.

Scholars have actively explored research related to high-quality development, mainly including: first, defining the connotation of high-quality development. Luo [25] argues that high-quality development implies a stable momentum of economic growth, greater achievements in coordinated regional development, and meeting the growing needs of the people. It can achieve multiple sustainable development goals of economic, social and environmental benefits [25–28]. Second, the indicator system is mainly constructed based on innovation [29], coordination [30], and sustainable development. Some preliminary studies have shown that the imbalance of high-quality development in service industry is prominent [3,14], with agglomeration effects [31–33]. The Yangtze River Delta region shows an inverted ’U’ shape of high-quality development, with significant regional differences [34,35]. These literatures make certain contribution to the field, with methods focusing on entropy weighting [36,37], Durbin model [34,38], Moran’s index [5,39]. Although these methods can reflect the level of high-quality development more accurately, they focus more on correlation analysis [40], and it is difficult to reflect the agglomeration effect and transfer regulations. In addition, the measurement of key indicators is mostly ignored or measured by weights [5], ignoring the complex influence relationship that exists between indicators.

High-quality development has regional differences [4,34,41] and there is an agglomeration and re-expansion effect in the service industry [6,42]. However, previous studies have usually explored these two in isolation, and the accuracy of the studies needs to be improved. Compared with the traditional methods mentioned above, Kernel density estimation, Dagum Gini coefficient and Markov chain can effectively deal with the problem of cross-overlap between samples [43]. It also reflects the dynamic evolution of the research object [44–46]. The reliability of this method has been demonstrated by numerous researchers in recent years [41,47,48]. Coupled with the fact that social network analysis (SNA) is able to quantitatively analyze various relationships [49,50], it provides new inspiration for this paper to explore the key factors (indicators).

Therefore, this paper took CCEC as an example and conducted research from the following aspects. First, the evaluation index system was constructed, and the high-quality development level of service industry was measured using the entropy method. Second, by using Kernel density estimation and Markov chain, the dynamic evolution characteristics and transfer patterns of service industry were analyzed. Third, Dagum Gini coefficient was used to measure spatial differences and identify sources of differences and their contributions. Then, the spatial characteristics of service industry agglomeration were analyzed by using the Koo method. Finally, the SNA was used to identify the core indicators. This paper aims to provide a certain decision-making basis for promoting the high-quality development of service industry and even the economy in the CCEC.

Marginal contribution of this paper: (1) Innovation of indicators. Based on the existing evaluation indicators, in order to better reflect the innovation and coordination of service industry. This paper complements indicators such as ’degree of industrial concentration’ and ’emergentization structure of service industry’. The aim is to assess the high-quality development of service industry in a more comprehensive way. (2) The Dagum Gini coefficient, Koo method, etc. were incorporated to measure regional differences and agglomeration characteristics of service industry. (3) The SNA method was innovatively introduced, providing a good foundation for determining the relationship between various factors and their internal roles.

The rest of the paper is organized as follows. The second section is the methods and materials. The third section presents the results. The last section is the discussion and conclusion.

## Materials and methods

### Study area

**Fig 1:**
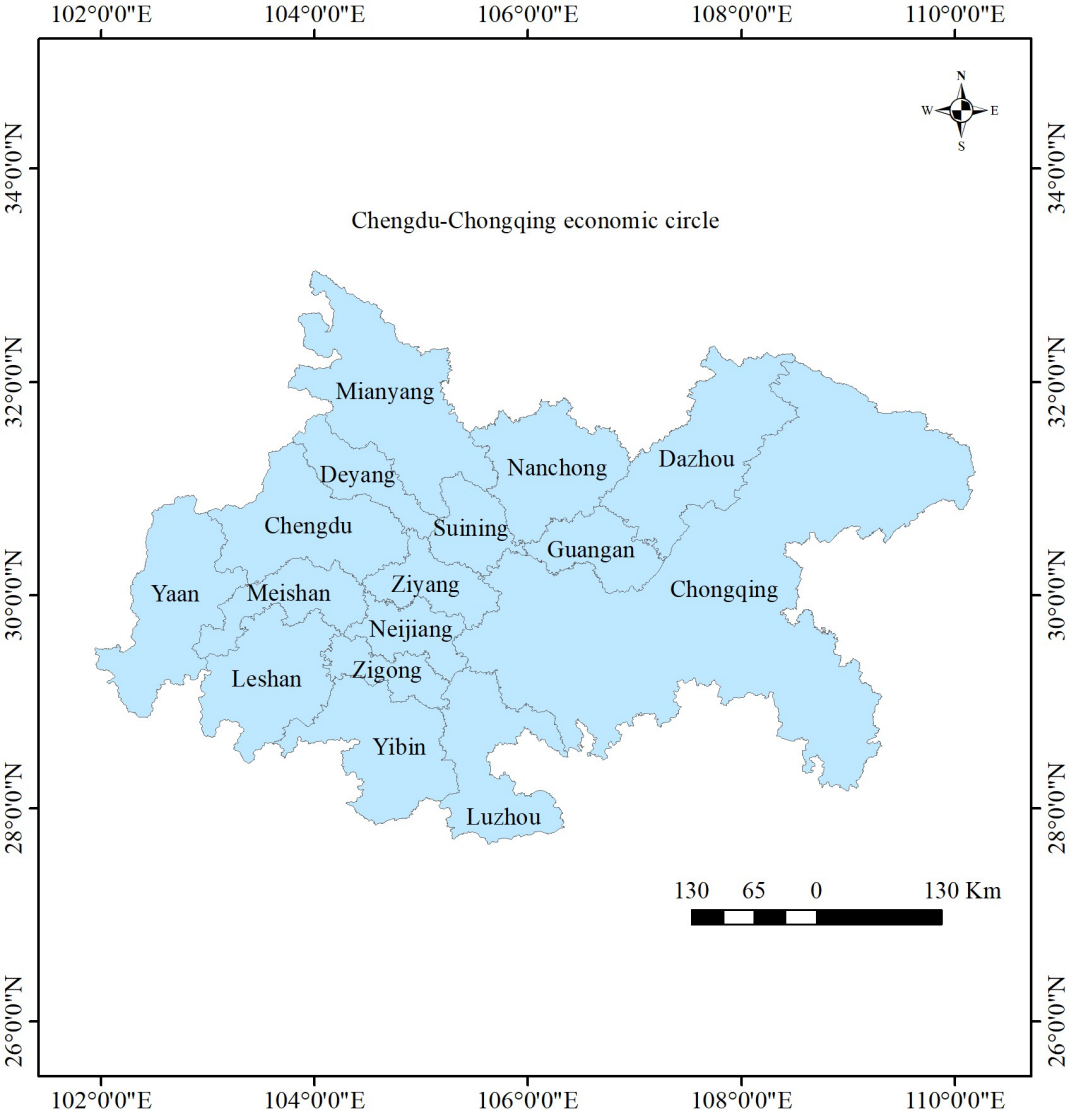
Study area. The base map outline was obtained by using ArcGIS 10.2 based on the Service Center of Standard Map (http://bzdt.ch.mnr.gov.cn/) and the permission number is GS (2016) 2923.

The CCEC (Fig 1) was first proposed in 2020, with a geographic range of 101°56’ ~ 109°15’E, 27°40’ ~ 32°19’N. The total area is 185000 square kilometers, including 27 districts (counties) in Chongqing and 15 cities in Sichuan Province [51,52]. The CCEC is divided into four urban agglomerations (metropolitan areas), as shown in Table 1. Located at the intersection of the Belt and Road and the Yangtze River Economic Belt, the CCEC is one of the important economic growth poles, and is the most economically developed region in southwest China [18]. The resident population of the CCEC in 2021 was 97.58 million people, and the GDP was 739.192 billion yuan [53]. The population density is high and the industrial base is the strongest [21].

**Table 1:** Urban agglomerations in the CCEC.

Urban agglomerations	Including cities
The Chengdu plain urban agglomeration	Chengdu, Mianyang, Deyang, Leshan, Suining, Yaan, Ziyang, Meishan
The northeast Sichuan urban agglomeration	Nanchong, Guangan, Dazhou
The south Sichuan urban agglomeration	Yibin, Luzhou, Zigong, Neijiang
Chongqing	Chongqing’s main urban area and 27 districts (counties)

### Evaluation index system for high-quality development of service industry

In the context of the new normal, the high-quality development of the service industry should not only continue to promote its internal development, but also emphasize coordination with the external system, such as the economy and society. Therefore, based on the inherent requirements of high-quality development [3,6,8,17,24,54–59], sustainable concept and the ’new development concept’ [2,5], this paper constructed an evaluation system that includes innovation, coordination, and sustainability as primary indicators (Table 2). Specifically, as follows: (1) Innovation. The innovative development of service industry is the first driving force for its high-quality development [3]. Based on the existing evaluation indicators, I1,…, and I4 [3,54] were selected as the innovation indicators. (2) Coordination. Coordination reflects the balance and interaction of service industry within (outside) the region [3]. Based on the existing literature [56], indicators such as C1 [3] and C2 [54] were selected. Inspired by scholar Cui (2022) [54], and taking into account the government’s emphasis on upgrading the industrial structure of service industry, ’emergentization structure of service industry’ (C4) was added. In addition, the fact that industrial development often has certain agglomeration and radiation effects was taken into account. Therefore, ’degree of industrial concentration’ (C3) was added. (3) Sustainability. Sustainability takes into account both the quality of supply of multiple factors in service industry and the stability of growth. Combined with existing research, S4 [3,55] can often reflect the stable growth of service industry and so on. In addition, some scholars have also suggested that S6 determines the ability of service industry to open up to the outside world [59]. Therefore, this study chose (S1) [24],…, (S7) [4,59] to reflect the sustainability of service industry.

**Table 2:** Evaluation index system for high-quality development of service industry.

Primary indicators	Secondary indicators	Tertiary indicators	Indicator sources
Innovation (I)	Innovative input	Science funding intensity (I1)	[3,24]
		Investment intensity in education (I2)	[55]
	Innovation output	Economic contribution of service industry (I3)	[3]
		Labour productivity of service industry (I4)	[3,54]
Coordination (C)	Regional coordination	Economic intensity of service industry (C1)	[3]
		Fixed assets investment level of service industry (C2)	[54,56]
	Industrial coordination	Degree of industrial concentration (C3)	[54]
		Emergentization structure of service industry (C4)	[54]
Sustainability (S)	Macroeconomic environment	Level of economic development (S1)	[24]
		Level of human capital (S2)	[4,24,38,56–58]
	Factor supply	Development of financial institutions (S3)	[24]
	Stability	Size of service industry (S4)	[55]
		Employment stability of service industry (S5)	[4]
	Facilities	Availability of transportation facilities (S6)	[24,34,57]
		Level of transportation production (S7)	[4,59]

### Method

#### Entropy weight method

Based on the result of data dimensionless, the entropy method is used for weighting [60], and the *H*_*index*_ is calculated.


$$H_{index}=\sum_{j=1}^{m}x_{ij}^{'}\omega_{j}$$ (1)

$x_{ij}^{'}$ is the result of the dimensionless processing of indicator by *x*_*ij*_. *ω*_*j*_ is the weight of the entropy method. *H*_*index*_ is the composite index of high-quality development of service industry.

#### Dagum Gini coefficient

Dagum Gini coefficient was chosen to describe the spatial evolution process of service industry. The detailed method calculation can be found in references [4,43].

#### Kernel density estimation

Kernel density estimation is a non-parametric estimation method, which is mainly used to estimate the probability density of random variables [34]. The formula is as follows.


$$f(x)=\frac{1}{Mh}\sum_{i=1}^{N}K(\frac{X_{i}-X_{a}}{h})$$ (2)

*M* is the number of observations. *h* is the bandwidth. *X*_*i*_ is the independent identically distributed observations. *X*_*a*_ is the observation mean. *K*(*x*) is the kernel function. In this paper, we used the Gaussian kernel density for estimation. The formula is as follows.


$$K(x)=\frac{1}{\sqrt{2\pi }}e^{\left(-\frac{x^{2}}{2}\right)}$$ (3)

#### Markov chain

The level of *H*_*index*_ is divided into N states through Markov chains, and a matrix consisting of N*N state transition probabilities is constructed. The transition probabilities can be described by the transfer matrix P [61,62].


$$P=(\begin{array}{ccc} m_{11} & \ldots & m_{1k}\\ \vdots & \ddots & \vdots \\ m_{k1} & \cdots & m_{kk} \end{array})$$ (4)

$$m_{ij}=\frac{n_{ij}}{n_{i}}$$ (5)

*n*_*ij*_ is the number of regions transferred from type *i* at moment *t* to type *j* at time *t+1*. *n*_*i*_ is the number of regions of type *i* at moment *t*.

#### Koo industrial agglomeration measurement method

The method proposed by J. Koo [63] was used to calculate the level of service industry agglomeration.


$$PC_{j}=\frac{E_{j}}{S_{j}\sum_{j=1}^{m}E_{j}}$$ (6)

*E*_*j*_ is the number of persons employed in service industry of city *j*. *S*_*j*_ is the total area of city *j*. $\sum_{j=1}^{m}E_{j}$ is the total employment in services in the country.

### Social network analysis

1. Overall network analysis

The overall network density and block analysis were used to measure the overall network.


$$The\;overall\;network\;density:\;\mathrm{ND}=\frac{K}{N(N-1)}$$ (7)

*K* is the total number of relationships (edges) in the network. *N* is the number of nodes.

The core idea of the block model is to divide the influencing factors in the network into several discrete subsets according to certain criteria, and to call these subsets ’blocks’, each of which is a subgroup of the overall network.

1. Individual network analysis

The individual network analysis is performed using betweenness centrality, degree centrality, and closeness centrality. The top five factors from each method have been processed in a concurrent set, which can provide key indicators for individual network analysis.


$$Betweenness\;centrality:\;BC=\sum_{j}^{n}\sum_{k}^{n}\frac{g_{jk}(i)}{g_{ik}}$$ (8)

*g*_*jk*_ is the number of shortcuts between node *j* and node *k*. *g*_*jk*_*(i)* is the number of shortcuts between node *j* and node *k* that exist through node *i*.


$$Degree\;centrality:\;\mathrm{DC}=\frac{k}{N-1}$$ (9)

$$Closeness\;centrality:\;\mathrm{CC}=\frac{N-1}{\sum_{j=1,j\neq i}^{n}d_{ij}}$$ (10)

*N*-1 is the maximum number of nodes connected to node *i*. *d*_*ij*_ is the distance value between node *i* and node *j*.

1. Identification of core indicators

**Fig 2:**
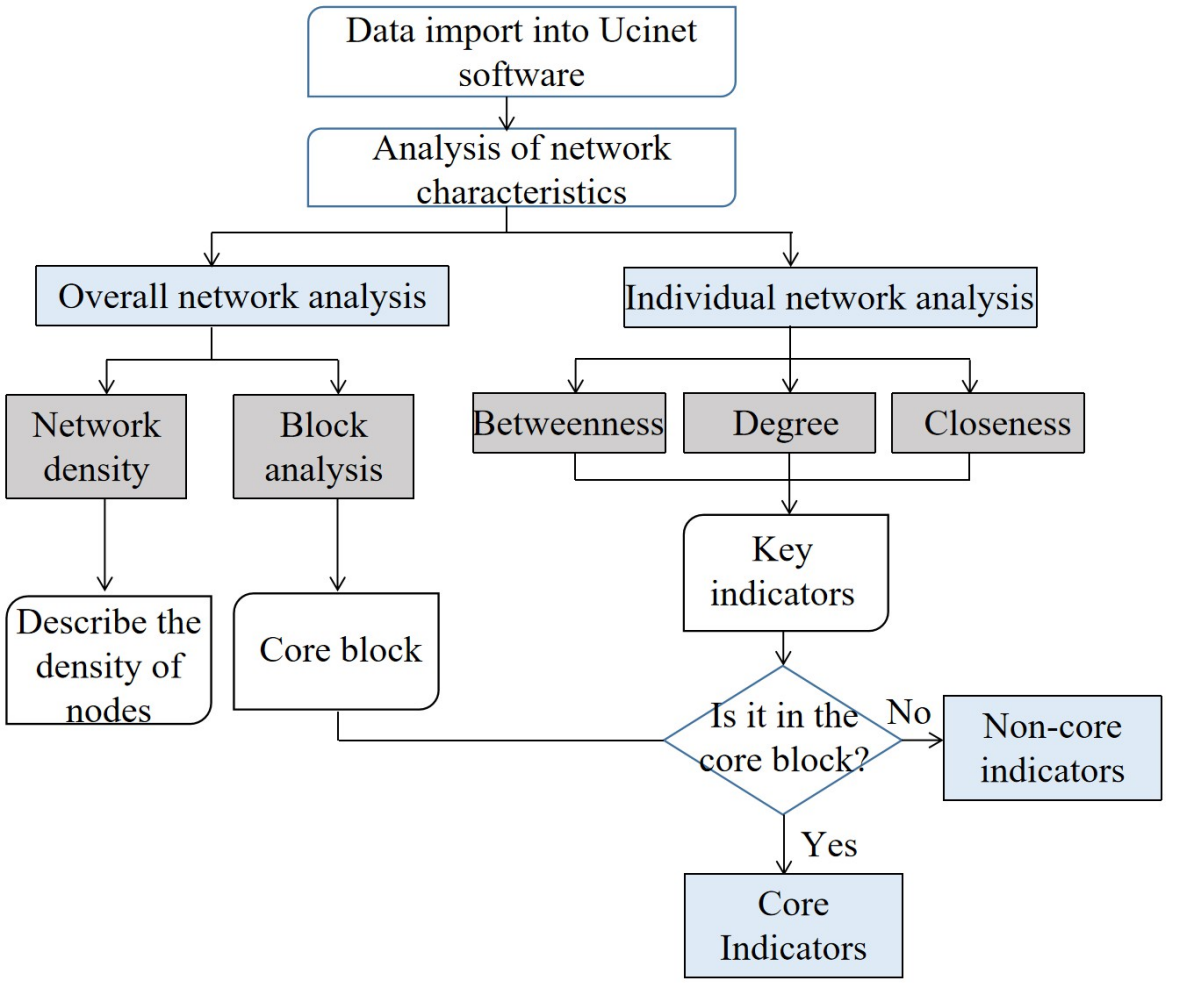
Framework of the SNA.

If the key indicators are in the core blocks, they are core indicators. The framework is shown in Fig 2.

### Data source and data processing

Due to the limitation on the length of this paper, the end years of the Tenth Five-Year Plan (2001–2005), the Eleventh Five-Year Plan (2006–2010), the Twelfth Five-Year Plan (2011–2015), and the Thirteenth Five-Year Plan (2016–2020) have been chosen as the scope of the study (they belong to an important five-year plan and are an important part of China’s national economic plan). That is, the four years 2005, 2010, 2015 and 2020 were selected to analyze high-quality development of service industry in the CCEC. In addition, due to the lack of statistical data at the county level, and in order to facilitate data collection and analysis, the study was based on the current practice of using data at the prefectural and municipal levels in general.

The processing of data for each variable is transformed as follows: I3 is expressed as ’value added of service industry/GDP’; C3 is represented by ’total output value of service industry/GDP’; C4 is expressed as ’employees in productive service industry/total employees in service industry’; S2 is expressed as ’number of persons with university or higher education/total employees’; S5 is represented by ’employees in service industry/total employees’; S6 is expressed as ’number of vehicles and operations at the end of the year’. The transformed data and other indicators can be directly obtained from the China Tertiary Industry Statistical Yearbook, the China Urban Statistical Yearbook, the China Economic and Social Big Data Research Platform and the CCEC Regional Statistical Yearbook (2006–2021). The data in the measurement model have been standardized to eliminate the influence of dimensionality.

## Results

### The *H*_*index*_ of service industry

*H*_*index*_ can be obtained by using the entropy weight method (S1 File). Based on the 2005–2020 *H*_*index*_ average (Table 3), the service industry in the CCEC is highly polarized, Chengdu has the highest *H*_*index*_, followed by Chongqing. The development level of service industry in the ’Chengdu and Chongqing’ dual core cities is relatively high, while the development level of the surrounding cities is relatively low. Compared with 2005, high-quality development level of service industry in Chengdu, Deyang, Mianyang, Leshan, Meishan, Guangan, Yaan, and Ziyang shows an increasing trend in 2020. *H*_*index*_ differences in the CCEC from 2005 to 2010, 2010 to 2015 and 2015 to 2020, account for 20.796–40.125% of the total *H*_*index*_ after excluding Chengdu and Chongqing city. And the total annual difference keeps increasing. Given Chongqing’s status as a municipality directly under the central government, these conclusions indirectly confirm the ’siphoning effect’ of Sichuan’s capital city (Chengdu) on other cities.

**Table 3:** *H*_*index*_ of service industry in the CCEC from 2005 to 2020.

	2005	2010	2015	2020	Average
Chongqing	0.778	0.773	0.560	0.669	0.695
Chengdu	0.713	0.802	0.669	0.823	0.752
Zigong	0.187	0.143	0.106	0.141	0.144
Luzhou	0.148	0.131	0.122	0.118	0.130
Deyang	0.146	0.138	0.120	0.189	0.148
Mianyang	0.156	0.198	0.135	0.217	0.177
Suining	0.095	0.096	0.068	0.053	0.078
Neijiang	0.135	0.091	0.067	0.130	0.106
Leshan	0.118	0.157	0.110	0.124	0.127
Nanchong	0.191	0.163	0.103	0.118	0.144
Meishan	0.096	0.083	0.235	0.139	0.138
Yibin	0.171	0.120	0.104	0.139	0.134
Guangan	0.100	0.150	0.279	0.123	0.163
Dazhou	0.189	0.144	0.088	0.134	0.139
Yaan	0.148	0.129	0.135	0.167	0.145
Ziyang	0.130	0.113	0.090	0.135	0.117

### Dynamic evolution characteristics of high-quality development in service industry

#### Dynamic characterization of the Kernel density estimation

**Fig 3:**
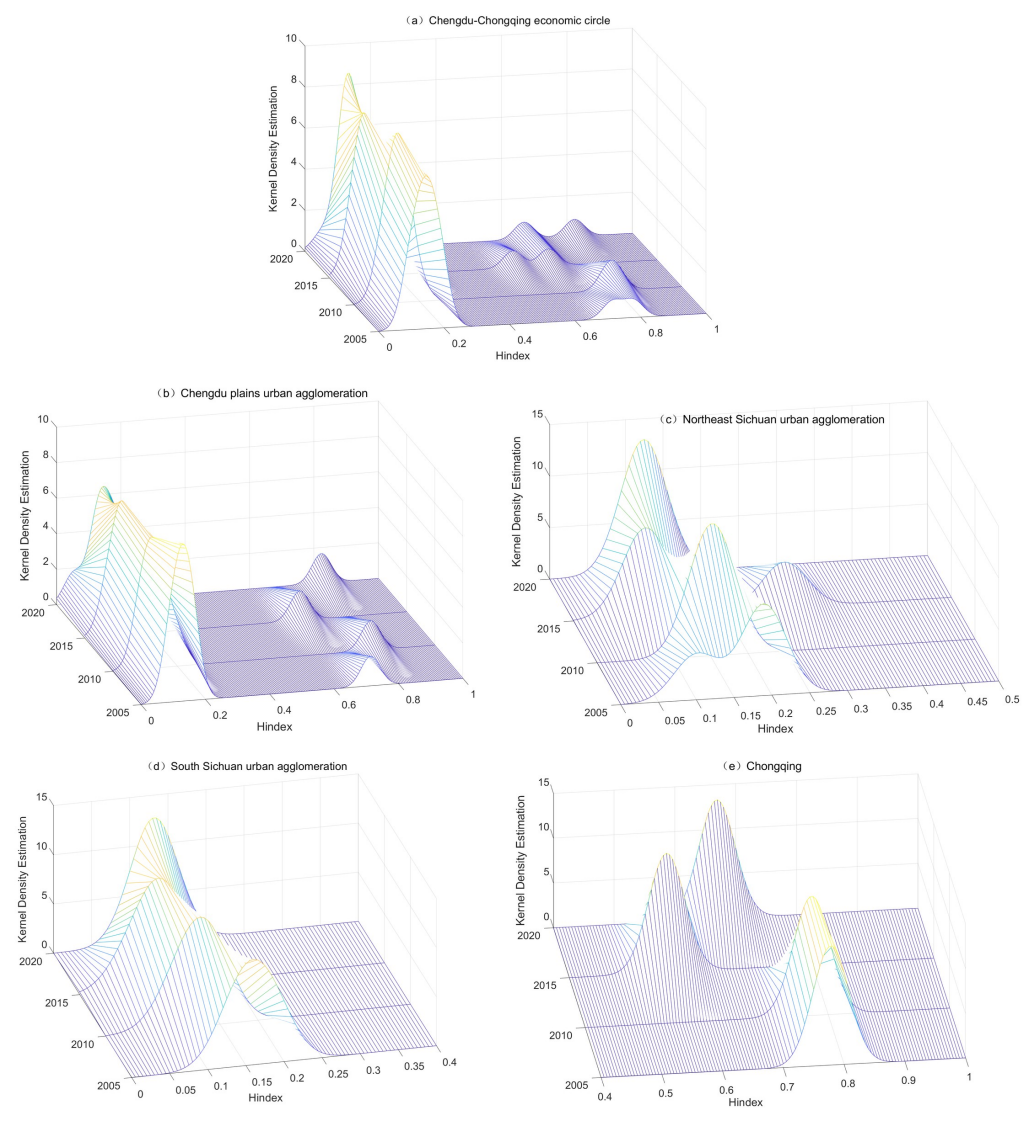
Dynamic evolution of high-quality development of service industry in the CCEC and four urban agglomerations.

In this paper, Kernel density estimation was used to analyze the characteristics of the distribution location, shape, ductility and polarization phenomenon of high-quality development in service industry. The results are shown in Fig 3.

Fig (3A) shows dynamic evolution of high-quality development of service industry in the CCEC. In terms of the distribution position, the centre of the overall distribution curve and the change interval have gradually shifted to the left, but the speed of movement has slowed down year by year, and the position of the wave peak is stable at 0.10–0.20. From the distribution pattern, the overall height of the main peak of the distribution curve shows an upward trend. At the same time, the width of the main peak has been shrinking, indicating that the level of high-quality development in the CCEC is tending towards concentration. In terms of distribution ductility, the curve has a right trailing phenomenon, and the gap within the CCEC has a tendency to widen. In terms of distribution polarization, the number of curve peaks has shifted from the ’main peak + right peak’ bimodal distribution pattern of 2005 and 2010 to a triple peak distribution. This indicates that high-quality development of service industry has changed from polarization to multi-polarization. And the phenomenon of polarization has also increased.

The Chengdu plain urban agglomeration (Fig 3B) is characterized by a double peak distribution of ’one main and one side’, and the distance between the two peaks is large. The main peak is located at 0.1–0.2, and the side peak is located at 0.5–0.8, and the side peak has a tendency to move to the left. This shows that there is an obvious polarization phenomenon in the Chengdu plain urban agglomeration. The northeast Sichuan urban agglomeration (Fig 3C) evolves from a ’trailing tail’ in 2005 to a ’single spike’ in 2020, indicating a weakening of the polarization effect of development in northeast Sichuan. There is only one main peak in the south Sichuan urban agglomeration (Fig 3D), and the position of the peak is more stable, ranging from 0.1 to 0.20. The amplitude of the change in the peak is small, but the curve changes from ’flat and wide’ in 2005 to ’steep and narrow’ in 2020 (the peak increases in width and narrows in width). This indicates that the degree of difference among the cities in the south Sichuan urban agglomeration has become smaller, and there is no polarization. The position of the main peak in Chongqing (Fig 3E) was in the range of 0.8–0.9 in 2005 and 2010, and between 0.55–0.7 in 2015 and 2020. The main peak as a whole experiences first a leftward and then a lateral movement, and all of them show single-peak characteristics without polarization.

#### Markov chain analysis

The Markov probability transfer matrix was constructed based on the measured *H*_*index*_ of the CCEC from 2005 to 2020, as shown in Table 4.

**Table 4:** Markov probability transfer matrix.

	Low level	Medium-low level	Medium-high level	High level	Observed value
Low level	0.400	0.333	0.200	0.067	15
Medium-low level	0.333	0.222	0.222	0.222	9
Medium-high level	0.333	0.333	0.167	0.167	12
High level	0.000	0.167	0.333	0.500	12

As can be seen from Table 4, the elements at the ends of the diagonal are larger than those in the middle of the diagonal. This indicates that cities with low and high levels of high-quality development of service industry have a relatively high probability of maintaining their initial level. Moreover, these cities have poor mobility and a solid size distribution, which makes it easy for them to fall into the ’low-level trap’ and ’high-level monopoly’. Second, the probability of upward transfers at the low, medium-low and medium-high levels is 33.3%, 22.2% and 16.7%, respectively. It can be seen that high-quality development of service industry is a dynamic process with twists and turns, and the development difficulties faced by different levels are different. Third, the probability of moving down one level for low, medium and high levels is 33.3% for all levels. This indicates that high-quality development of service industry in the CCEC is prone to instability, and there is a certain risk of downshifting. Therefore, each urban agglomeration should be alert to the risk of downward level transfer. Taking care to prevent the achievements of service industry from ’regressing’. And maintain the stability of existing development achievements and strive to achieve upward level transfer.

### Analysis of regional differences in service industry

Kernel density can capture the dynamic evolution trend of service industry, but it cannot capture the relative change in the spatial difference between the industry and its source. The Dagum Gini coefficient can compensate for this shortcoming by providing a more detailed picture of the spatial evolution of the service industry from the perspective of relative differences. The decomposition results of Dagum Gini coefficient are shown in Table 5.

**Table 5:** Regional differences and decomposition results.

		2005	2010	2015	2020
Overall Gini coefficient		0.366	0.394	0.417	0.380
Breakdown and contribution	Intra-regional differences	0.091	0.107	0.112	0.113
	Contribution rate (%)	24.851	27.237	26.940	29.625
	Inter-regional differences	0.200	0.251	0.247	0.239
	Contribution rate (%)	54.611	63.664	59.302	62.959
	Hypervariable density	0.075	0.036	0.057	0.028
	Contribution rate (%)	20.538	9.099	13.759	7.416
Intra-regional differences	Ⅱ	0.369	0.414	0.402	0.409
	Ⅲ	0.070	0.086	0.103	0.038
	Ⅳ	0.125	0.029	0.270	0.029
Inter-regional differences	Ⅰ-Ⅱ	0.591	0.573	0.548	0.530
	Ⅰ-Ⅲ	0.658	0.728	0.741	0.671
	Ⅰ-Ⅳ	0.659	0.671	0.620	0.686
	Ⅱ-Ⅲ	0.282	0.340	0.367	0.336
	Ⅱ-Ⅳ	0.317	0.309	0.376	0.352
	Ⅲ-Ⅳ	0.123	0.113	0.280	0.044

Note: As Chongqing municipality is treated as a separate group, the analysis of intra-regional differences is not applicable. Ⅰ, Ⅱ, Ⅲ and Ⅳ denote Chongqing, Chengdu plain urban agglomeration, Sichuan south urban agglomeration and Sichuan northeast urban agglomeration, respectively.

The overall Gini coefficient in the CCEC shows a fluctuating trend of first increasing and then decreasing (rapid increase from 2005 to 2010, slow rise from 2010 to 2015 and decline from 2015 to 2020). This indicates that the regional differences in the of service industry first increase and then decrease.

1. Intra-regional differences. Overall, intra-regional differences are small, with a downward trend after a brief increase in 2015. Among them, the northeast Sichuan urban agglomeration has the largest intra-regional differences, while the Chengdu plain urban agglomeration has the smallest intra-regional differences. And the Chengdu plain urban agglomeration is ahead of other regions in terms of high-quality development. Specifically, the average intra-regional Gini coefficient for the Chengdu plain urban agglomeration is 0.399, with the smoothest change from 0.369 in 2005 to 0.409 in 2020. In contrast, in 2020, the Gini coefficients of the south Sichuan urban agglomeration and the northeast Sichuan urban agglomeration are lower than their respective averages, with decreases of 46.59% and 76.97%, respectively.
2. Inter-regional differences. The inter-regional differences in the Chongqing-Chengdu plain (Ⅰ-Ⅱ), Chongqing-south Sichuan (Ⅰ-Ⅲ), and south Sichuan-northeast Sichuan (Ⅲ-Ⅳ) show a significant fluctuating downward trend. The inter-regional differences between Chengdu plain-south Sichuan (Ⅱ-Ⅲ) and Chengdu plain-northeast Sichuan (Ⅱ-Ⅳ) show a slight increase, but the general trend is a slow fluctuating decrease. The inter-regional differences between Chongqing-northeast Sichuan (Ⅰ-Ⅳ) are on increasing. On the other hand, the largest inter-regional difference is in Chongqing-south Sichuan (Ⅰ-Ⅲ). The gap between south Sichuan-northeast Sichuan (Ⅲ-Ⅳ) fluctuates, but is always the smallest inter-regional difference. This indicates that the level of service industry development in the urban agglomerations of south Sichuan and northeast Sichuan is comparable. After 2015, except for Chongqing-northeast Sichuan (Ⅰ-Ⅳ), where the Gini coefficient of inter-regional differences increased (regional gap widening), all other regional inter-regional differences decreased (regional gap narrowing). Overall, the gap between urban agglomerations has gradually narrowed, and service industry has tended to be coordinated between regions.
3. The contribution rate of inter-regional differences is the largest, always remaining above 54.61%. The average contribution rate of hypervariable density is 12.7%, indicating that the crossover problem of samples has little impact on the differences in high-quality development in the CCEC. Therefore, spatial differences are mainly due to the differences between four regions. In the future, how to reduce inter-regional differences in service industry development will be an important direction for promoting coordinated and high-quality economic development.

### The agglomeration level of service industry

According to the Koo method, the level of service industry agglomeration in the CCEC from 2005 to 2020 can be obtained, as shown in Table 6.

**Table 6:** Agglomeration level of service industry in the CCEC.

	2005	2010	2015	2020
Chengdu plain urban agglomeration	0.399	0.388	0.396	0.397
Chongqing	0.041	0.042	0.042	0.039
South Sichuan urban agglomeration	0.187	0.189	0.180	0.156
Northeast Sichuan urban agglomeration	0.104	0.106	0.109	0.109
CCEC	0.731	0.725	0.727	0.701

**Fig 4:**
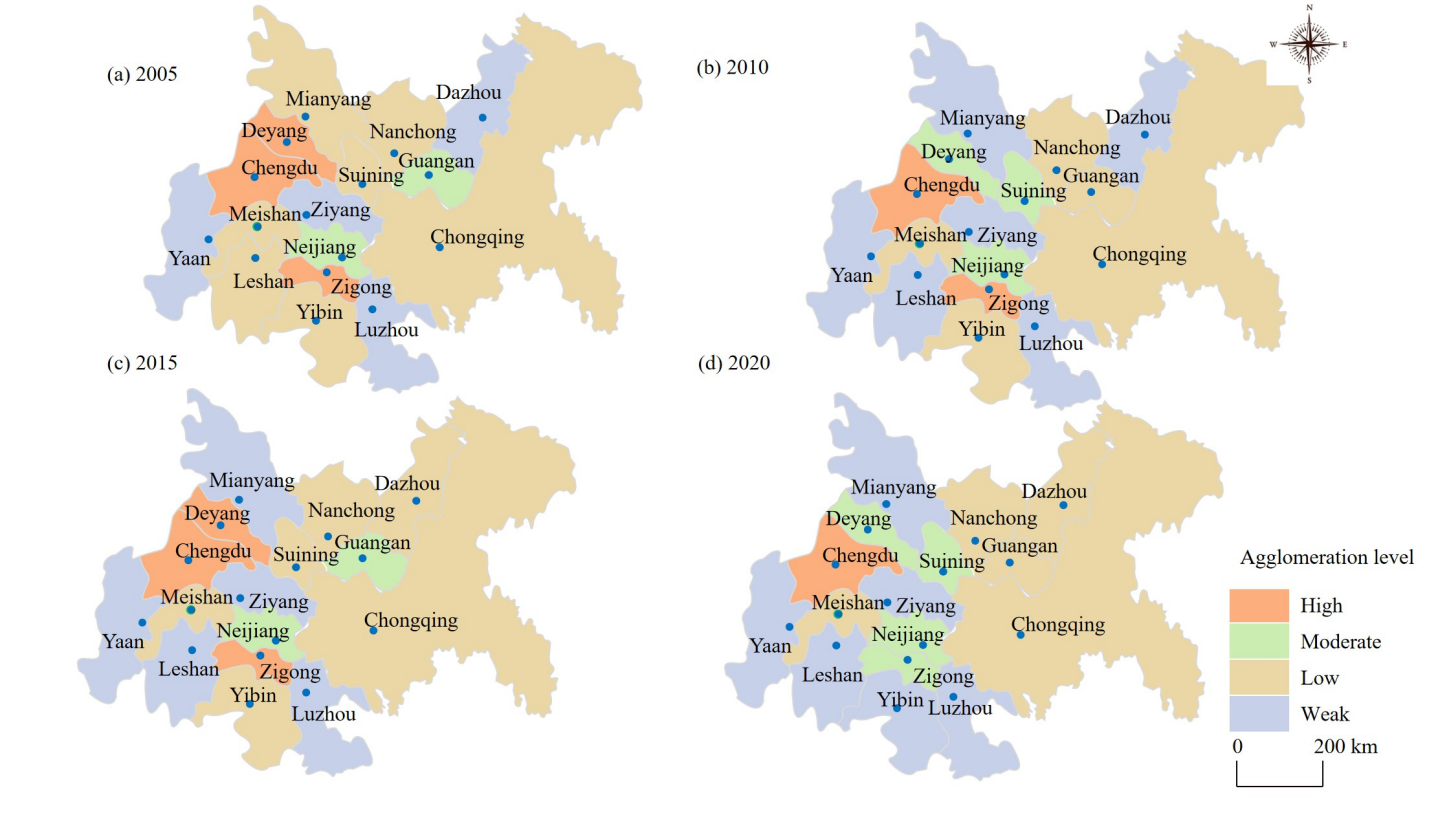
Spatial divergence of service industry agglomeration in the CCEC. Source: Created by the author based on the base map of the CCEC, which comes from the Service Center of Standard Map (http://bzdt.ch.mnr.gov.cn/), and the number of the permission is GS (2016) 2923.

From 2005 to 2020, the agglomeration level of service industry in the CCEC has little difference and shows a convergent decline. Among them, the main reason for the difference is that the agglomeration level of service industry in south Sichuan decreases from 0.187 in 2005 to 0.156 in 2020. The rest of the regions fluctuate in a smaller range. In order to distinguish the spatial distribution characteristics of service industry agglomeration in a more refined way, the spatial divergence map of agglomeration is drawn (Fig 4).

Overall, the level of agglomeration in the CCEC is relatively low during the study period, and the spatial imbalance did not show a continuous increase. In terms of geographical distribution, weak agglomeration is mainly dispersed in the western part of the CCEC, with the lowest level of agglomeration in Yaan City. The highly agglomerated cities include Chengdu and Deyang. And from 2005 to 2020, the city with a high agglomeration level (Chengdu) shows a strong capacity for solidity and maintains a high level of agglomeration. In a horizontal comparison, the change in the agglomeration level of each city is small, and it is a gradual development (not a leapfrog development). By comparing the five cities with the highest (lowest) agglomeration level, the agglomeration level tends to stabilize. These cities show a converging upward (downward) trend, indicating that service industry agglomeration has a certain self-reinforcing and stabilizing function.

### Identification results of core indicators

As shown earlier in this paper, there are significant spatial differences in the dynamic evolution of high-quality development of service industry in the CCEC. These differences were analyzed based on the evaluation indicators, but the degree of influence between indicators was overlooked. Therefore, it is necessary to extract the core indicators. To this end, this paper surveys seven experts in the research fields of regional economy and industrial economy (S1 Table) to evaluate the indicator matrix. This is done as follows: the indicator matrix is constructed entirely on the basis of the indicator system (Table 2), and the indicators in the rows of the matrix are compared two by two with the indicators in the columns. If an influence relationship is considered to exist, 1 is selected, otherwise it is 0.

**Fig 5:**
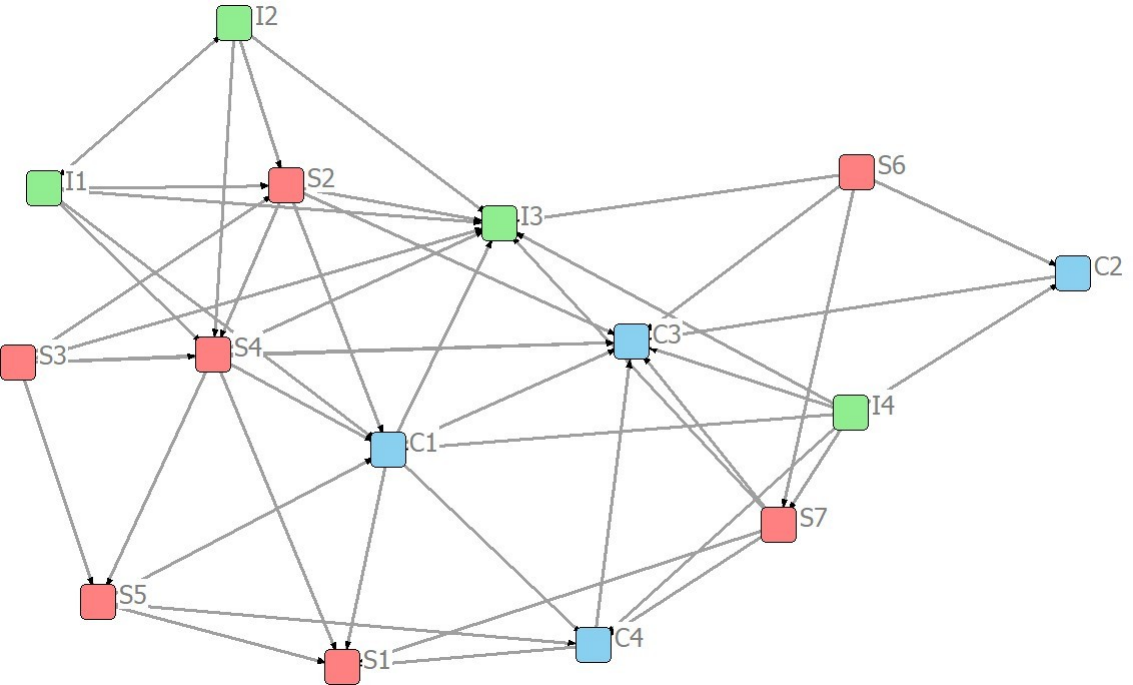
Network visualization of indicators.

**Fig 6:**
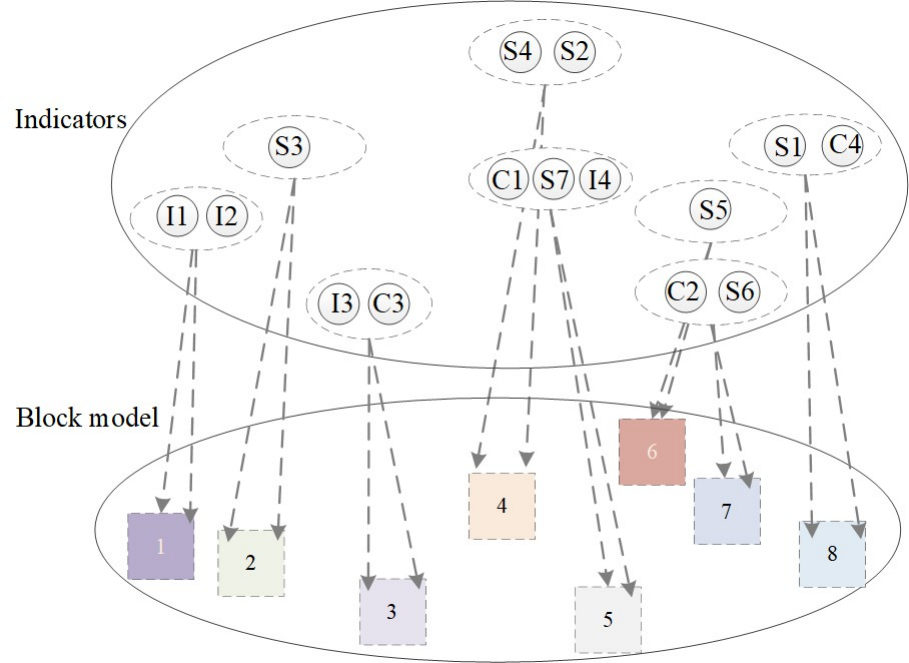
Block of indicators.

(1) Consensus Analysis
  Consensus analysis was used to verify whether the seven experts were consistent in their answers to the questions. The Ucinet software gives a ’ratio of largest to next’ of 13.327 in the consistency matrix, which is greater than 3. This indicates that the collected data is homogeneous and can be used for further calculations and research.
Overall network analysis
  The network was visualized using Netdraw software, as shown in Fig 5. Using Ucinet software, the number 3 was chosen as the maximum depth of splits (not blocks) to produce a chunked plot for each metric (Fig 6) and a density matrix (Table 7).

**Table 7:** Density matrix.

	Block 1	Block 2	Block 3	Block 4	Block 5	Block 6	Block 7	Block 8
Block 1	0.143	0	0.071	0.893	0.024	0	0	0
Block 2	0	0	0.571	1	0	1	0	0
Block 3	0	0	0	0	0	0	0	0
Block 4	0	0	0.143	0.143	0.048	0.214	0	0.25
Block 5	0	0	0.31	0	0.119	0	0.024	0.714
Block 6	0	0	0	0	0.048	0	0	0.714
Block 7	0	0	0.536	0	0.071	0	0.071	0
Block 8	0	0	0.25	0	0	0	0	0.214

This paper converted the density matrix into the image matrix. The overall network density value was found to be 0.1129. This paper replaced the value of 0.1129 in Table 7 with 1 and vice versa with 0, the image matrix was obtained as shown in Table 8.

**Table 8:** Image matrix.

	Block 1	Block 2	Block 3	Block 4	Block 5	Block 6	Block 7	Block 8	Sending relationship	Relationship to self
Block 1	1	0	0	1	0	0	0	0	1	1
Block 2	0	0	1	1	0	1	0	0	2	0
Block 3	0	0	0	0	0	0	0	0	0	0
Block 4	0	0	1	1	0	1	0	1	3	1
Block 5	0	0	1	0	1	0	1	1	3	1
Block 6	0	0	0	0	0	0	0	1	1	0
Block 7	0	0	1	0	0	0	1	0	1	1
Block 8	0	0	1	0	0	0	0	1	1	1
Receiving relationship	0	0	7	2	0	2	1	3		
Relationship to self	1	0	0	1	1	0	1	1		

According to Burt’s theory of positional division [64], block 1, block 2, block 3 and block 5 are in isolated positions. Where block 1, block 2 and block 5 have only a sending (influence transfer) relationship, and block 3 has only a receiving relationship. Blocks 4, 7 and 8 are in the ’primary person’ position (both sending and receiving relationships, and strong internal connections). Block 6 is in the ’broker’ position (both sending and receiving relationships, but no internal connections). Nodes at the center of the network should have both sending and receiving relationships, so blocks in the ’primary person’ and ’broker’ positions are likely to be at the center.

1. Individual network analysis

The degree centrality, closeness centrality and betweenness centrality of the network were calculated as shown in Table 9.

**Table 9:** Individual network analysis results.

	Betweenness	Degree	Closeness
I1	0.583	2	58.333
I2	0	2.286	53.846
I3	0	1.286	73.684
I4	0	2.286	63.636
C1	6.417	3.143	73.684
C2	0	1.286	48.276
C3	0	5.714	73.684
C4	0.833	5.429	60.87
S1	0	3.143	58.333
S2	1.75	3.714	66.667
S3	0	4.143	60.87
S4	7.5	4.714	73.684
S5	2.583	3	56
S6	0	1.714	56
S7	2.333	3.286	63.636

Degree centrality is the number of other indicators that are directly connected to a particular indicator. A higher value of degree centrality indicates that the node (indicator) is significantly connected to other nodes (indicators). The average value of centrality for the whole network is 3.143, and indicators C3, C4, S4, S3, S2, and S7 have higher than average centrality, indicating that these factors have high relevance and priority in the network. Closeness centrality considers the average length of the shortest distance from each node to other nodes. The range of proximity centrality is [48.276, 73.684], indicating that each indicator can be connected to other indicators relatively quickly. And the proximity centrality of the indicators C3, C1, I3, S4, S2, S7, and I4 is above the mean (62.747), which indicates that the closer the connection to other nodes. Betweenness centrality reflects the proportion of nodes on all the shortest paths. The higher the value, the more important the node is. The sum of the betweenness centrality of S4, C1, S5, S7 and S2 accounts for 93% of the total number of nodes, and they are the key nodes in the network.

The top five indicators ranked by betweenness centrality are C3, C4, S4, S3 and S2. The important indicators derived from degree centrality are C3, C1, I3, S4 and S2, and the top five indicators ranked by closeness centrality are S4, C1, S5, S7 and S2. Therefore, the key indicators obtained by individual network are S4, S2, C3, C4, S3, C3, C1, I3, C1, S5 and S7.

(4) Core indicators

S4 and S2 belong to block 4, S5 belongs to block 6, and C4 belongs to block 8, which belongs to the core block in the overall network analysis (Fig 6). And the other key indicators do not belong to the core block. Combined with the identification criteria in Fig 2, the core indicators are: level of human capital (S2), size of service industry (S4), employment stability of service industry (S5), and emergentization structure of service industry (C4).

## Discussion

The contribution of this paper is twofold. First, in terms of measurement methods, unlike traditional research, this paper not only applies the Kernel density method, Markov chain and Dagum Gini coefficient to analyze the regional differences, but also further integrates the Koo method to explore the agglomeration effects of service industry. Second, the key factors affecting the high-quality development of service industry are explored from the perspective of internal driving factors. Unlike existing studies that mostly measure the importance of indicators based on weight, this paper adopts the SNA method to identify the core factors. In this way, this paper provides a framework for analyzing the service industry that integrates spatio-temporal analysis and key factor extraction.

### Time difference analysis

This study comprehensively evaluates the high-quality development level of the CCEC’s service industry. In many countries, with increasing economic globalization and a deepening regional division of labour, the demand for service industry has gradually expanded. This demand has led to an uneven development of service industry, which is reflected in changes in the value added of the service industry, kernel density and the Dagum Gini coefficient. In terms of time differences, the *H*_*index*_ fluctuations do not have the same characteristics (Table 3). The CCEC presents the ’Chengdu and Chongqing’ twin core cities [19], which have a high level of *H*_*index*_. This finding is consistent with existing studies [51], which is rare for cities in the western region. However, other neighboring cities have a lower *H*_*index*_. Comparing the 2020 and 2005 values, Chengdu, Deyang, Mianyang, Leshan, Meishan, Guangan, Yaan, and Ziyang show growth in the level of high-quality development of service industry.

From Fig 3(A)–3(E), it can be seen that the polarization effect of the CCEC has increased, and the internal gap tends to widen over the years [20]. And the characteristics of changes in Chengdu plain and the CCEC are similar (time dimension). The development of service industry was uneven from 2005 to 2020. The Kernel density estimation curves for the northeast Sichuan (Fig 3C), the south Sichuan (Fig 3D), and Chongqing area (Fig 3E) do not show an increase in the horizontal width of the kurtosis or an evolution towards ’multiple peaks’ when comparing 2020 with 2005. This suggests that the gap between these three urban agglomerations (metropolitan areas) is stable over time, with no significant polarization [10].

From the Dagum Gini coefficient, the overall Gini coefficient shows an upward trend of continuous increase during 2005–2015. There is reason to suspect that this may be due to the polarization effect of the provincial capitals (with the largest intra-regional differences in the Chengdu plain urban agglomeration), which leads to greater regional differences in service industry development. In 2020, the value becomes smaller, suggesting that regional differences in high-quality development of service industry are narrowing. This may be due to the upgrading and restructuring of service industry, or it may be affected by the Corona Virus Disease 2019.

### Spatial difference analysis

Influenced by the differences in human resources and the unevenness of spatial changes, etc., high-quality development of service industry in the CCEC shows obvious spatial differentiation and dynamic evolution characteristics. Among the regional differences, inter-regional differences make the largest contribution, consistently remaining above 54.61%. This is consistent with the findings of existing studies [3], and different from the findings of existing studies, which suggest ’the contribution of hypervariance density is the main reason for the large gap in high-quality development’ [35]. It can be seen that the CCEC should pay more attention to the spatial imbalance of inter-regional development in order to reduce inter-regional development differences. Despite the differences in contribution rates between different urban agglomerations, the identification of dominant contributions and sources of differences is stable in this study. Among them, the Chongqing-south Sichuan urban agglomeration has consistently had the largest inter-regional differences over the study period.

In terms of the spatial agglomeration, the spatial imbalance of the service industry has not shown any sustained increase. Weak agglomeration region is mainly concentrated in the western part of the CCEC. In particular, this study find that the spatial distribution of service industry agglomeration is not consistent with the *H*_*index*_ in general. The ’agglomeration effect’ of Chengdu is significantly stronger than that of Chongqing during the study period, which is basically in line with the existing conclusion [14]. The conclusion of this paper confirms that in the process of service industry development, there is a characteristic of agglomeration towards strong and developed cities with good industrial environments [58]. This expands the understanding of the ’Chengdu and Chongqing’ twin core cities in the CCEC. Due to the limited market size, compared with many generally well-developed urban agglomerations [11,12], the agglomeration and rapid expansion of the core cities in the CCEC has inevitably weakened the service clustering capacity of the surrounding cities [20]. This also shows that the constraints of administrative boundaries still exist, although the CCEC’s regional government has continued to reduce administrative barriers to economic development [12]. Second, as the capital city of Sichuan province, the strong ’siphon effect’ of Chengdu remains prominent.

### Analysis of core indicators

Previous studies have shown that there are many factors affecting the high-quality development of service industry, and the entropy method is mostly chosen to measure the importance of influencing factors [5]. However, the internal connections between factors and the extraction of key factors are neglected [3]. The results show that the degree of connection and closeness between nodes (factors) and other nodes vary in the SNA analysis. Specifically, from the results of indicator extraction, the key indicators and core indicators are not identical. However, S2, S4, S5 and C4 are important factors affecting the high-quality development of service industry. This finding will be useful for relevant government departments in the CCEC to make decisions. For example, in order to further improve the high-quality development level of service industry, it is necessary to focus on these indicators.

### Future research

This article analyzes regional differences in the high-quality development of CCEC’s service industry from different perspectives. However, this study has not fully explored its driving mechanisms, which could serve as a direction for further research.

## Conclusion

Taking the CCEC as an example, based on various measurement methods, this study explores the regional differences and key factors of the high-quality development of service industry. The main conclusions are as follows:

1. There are significant differences in the high-quality development of service industry in the CCEC. *H*_*index*_ is higher in Chengdu and Chongqing than in other regions. From a temporal perspective, the overall *H*_*index*_ first decreases and then increases. This trend is due to the transformation of traditional service industry and the popularity of modern services, as well as the consumption of resources. Therefore, the development of service industry should consider the coordination of multiple industries.
2. The development of service industry in the CCEC is uneven, with the Matthew effect and stratification. The level of agglomeration in northeast Sichuan achieves convergent development in 2020. However, in 2005–2020, the spatial agglomeration in the Chengdu plain and south Sichuan shows obvious stratification characteristics. Therefore, it is necessary to play the driving role of advantageous cities and promote the coordinated development of service industry in multiple regions.
3. The high-quality development level varies according to the existing foundation of service industry. When at a low level, there is a greater possibility of upgrading to the next level. As the grade increases, the level of service industry is unstable. Therefore, the CCEC’s governments should avoid regressing the development results of service industry. It also needs to establish a new system and experience sharing mechanism to achieve stable and efficient development of service industry.
4. This article also verifies that factors such as the level of human capital and employment stability are intrinsic driving forces for the high-quality development of service industry. It confirms that talent is an important core for the sustainable development of service industry. Improving the quality of labor force contributes to the stability of service industry.

## Supporting information

S1 File Standardization and weighting of indicators. (XLSX)

S1 Table Professional background of seven experts. (XLS)
